# Are insects declining and at what rate? An analysis of standardised, systematic catches of aphid and moth abundances across Great Britain

**DOI:** 10.1111/icad.12412

**Published:** 2020-03-04

**Authors:** James R. Bell, Dan Blumgart, Chris R. Shortall

**Affiliations:** ^1^ Rothamsted Insect Survey, Rothamsted Research, West Common Harpenden UK

**Keywords:** Aphids, insect conservation, light traps, mgcv, moths, poptrend, suction traps

## Abstract

1. Although we have known anecdotally that insects have been declining in Great Britain for more than 100 years, insect declines have only been statistically estimated over the last 20 years. Estimation of the rate of those declines is still hotly debated, fuelled by a lack of standardised, systematically collected data.
2. More than 24 million individual moths and aphids collected from 112 light traps and 25 12.2 m suction‐traps, respectively, were analysed using mixed models. Our objective was to estimate the long‐term trends in both groups based on annual totals recorded every year between 1969 and 2016.
3. The models showed that two paradigms existed: Over 47 years, long‐term linear trends showed that moths had declined significantly by −31%, but short‐term trends indicated that there were periods of significant decline and recovery in most decades since the 1960s. Conversely, despite aphid annual totals fluctuating widely, this group was in a steady state over the long‐term, with a non‐significant decline of −7.6%. Sensitivity analysis revealed that moth trends were not driven by a group of abundant species, but the sign of the overall aphid trends may have been driven by three of the most abundant species.
4. The spatial extent of moth trends suggests that they are extremely heterogeneous. Uniquely, moth declines were different among several habitat types, with robust significant declines found in coastal, urban and woodland habitats, but notably not in agricultural, parkland and scrubland habitats. Conversely, aphid trends showed spatial synchrony extending to 338 km, albeit with local variation.

## Graphical abstract

Graphical abstract

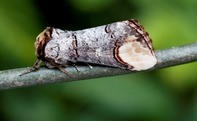

- Whilst the great majority of scientists agree that insects are in decline, there is much debate over the rate of decline, fuelled by a lack of standardised, systematically collected data.
- The Rothamsted Insect Survey has the most comprehensive, standardised long‐term data on insects in the World. Here, we present an analysis of moth and aphid population linear and non‐linear trends.
- We show, using 24 million individuals over 47 years, that moths have significantly declined by 31%, with differences between habitats. Aphids as a group were not in significant decline.

## Introduction

Recently, there has been a flurry of insect decline papers. Leather (2018) has expressed some bemusement as to why Hallmann *et al*. (2017) and Sánchez‐Bayo and Wyckhuys (2019) had received widespread media attention, given that insect declines have been apparent for decades. Indeed, nearly 50 years ago, Taylor stated that there had been a dramatic decline in moth populations between 1940 and 1960 (Taylor, 1974). Even earlier, Ford (1945, 1955) stated that many moths and butterflies had become scarce after the 1850s. Although neither of these studies report a rate of decline, they are indicative of widespread and dramatic change in both the 19th and early 20th century moth populations. Recently, insect declines have received rigorous analyses of historical time series. Conrad *et al*. (2006) echoed Taylor's concerns, highlighting a decline in two‐thirds of Britain's larger moth species between 1968 and 2002. A re‐analysis of more recent data (1968–2007) showed that 37% of the 337 species decreased by at least 50%, although those declines were largely clustered in southern Britain (Fox *et al*., 2013). Negative trends were shown for native bees in both Britain and the Netherlands (Biesmeijer *et al*., 2006) and Brooks *et al*. (2012) reported declines in British carabid populations of 28–52%. Both Warren *et al*. (2001) and Thomas *et al*. (2004) also showed declines in the distribution of butterflies, the latter study by as much as 74%. Butterflies are one of the best studied groups and it is estimated that their abundance globally has declined by 35% over 40 years (Dirzo *et al*., 2014) with potentially steeper declines in the Netherlands, estimated using non‐standardised techniques (van Strien *et al*., 2019). Even though we appear to have compelling evidence of declines, Thomas *et al*. (2004) asserted that we know very little about the state of insect populations beyond Europe and North America. This geographical bias has emerged as a major issue in recent global assessments (Simmons *et al*., 2019; Thomas *et al*., 2019).

The Sánchez‐Bayo and Wyckhuys (2019) paper that reviewed declines and the associated media stories are not without their critics, highlighting many issues around geographical, taxonomic and methodological biases that have purportedly undermined both the peer review process and insect conservation efforts (Leather, 2018; Cardoso *et al*., 2019; Mupepele *et al*., 2019; Saunders, 2019; Simmons *et al*., 2019; Thomas *et al*., 2019; Wagner, 2019). Yet, at the level of Insecta, many scientists would agree anecdotally that insect declines have happened in their lifetime, at least in many parts of the world (McCarthy, 2015: Vogel, 2017; Janzen & Hallwachs, 2019), and instead the dispute is likely around the rate rather than the existence of a decline for most groups.

Previous multi‐species studies on insect groups including moths, butterflies, hoverflies and carabid beetles showed that while most species are declining, there are subsets of species within each group that are increasing (Warren *et al*., 2001, Brooks *et al*., 2012, Fox *et al*., 2013) or at least remaining stable over time (Biesmeijer *et al*., 2006; Shortall *et al*., 2009; Hallmann *et al*., 2020). Even after the widespread habitat destruction during World War I, it is perhaps surprising that butterflies and moths saw a reversal in fortunes, although there are exceptions (Bretherton, 1951). Whilst it is rarely recognised or cited that insects may profit as a result of environmental change (Bell *et al*., 2015; Herrera, 2019, Boyes *et al*., 2019, Macgregor *et al*., 2019), it adds a dimension to the question of whether insects are declining and if so at what rate, because it suggests that to answer such a question will be dependent on the species or group studied, ignoring other likely important covariates such as habitat, spatio‐temporal issues, statistical methodology and sampling intensity and bias, for example (McKinney, 1999; Cardoso *et al*., 2019; Mupepele *et al*., 2019; Simmons *et al*., 2019; Thomas *et al*., 2019).

The ‘rate debate’ has been fuelled because of a lack of standardised data (Saunders 2019; Didham *et al*., 2020; Montgomery *et al*., 2020). To accurately capture the underlying trend in the rate of change requires simultaneously and repeatedly sampling populations with standardised devices (New, 1998). Since 1964, the Rothamsted Insect Survey has been at the forefront of the insect declines research, exploiting the longest standardised terrestrial insect time series in the world, reporting on population change in aphids, moths, ladybirds, wasps and general insect biomasses (Taylor, 1974; Conrad *et al*., 2002, 2004, 2006, Fox *et al*., 2006; Conrad *et al*., 2007; Woiwod & Gould, 2008; Shortall *et al*., 2009; Fox *et al*., 2013; Comont *et al*., 2014; Bell *et al*., 2015; Lester *et al*., 2017; Martay *et al*., 2017; Coulthard *et al*., 2019; Dennis *et al*., 2019; Fox *et al*., 2019; Macgregor *et al*., 2019). In this article, we build on this extensive knowledge and present an analysis of the likely scale of moth and aphid population linear and non‐linear trends over Great Britain and, how rates of change vary according to habitat and spatial scale.

## Methods

### *Sampling*

**Figure 1:**
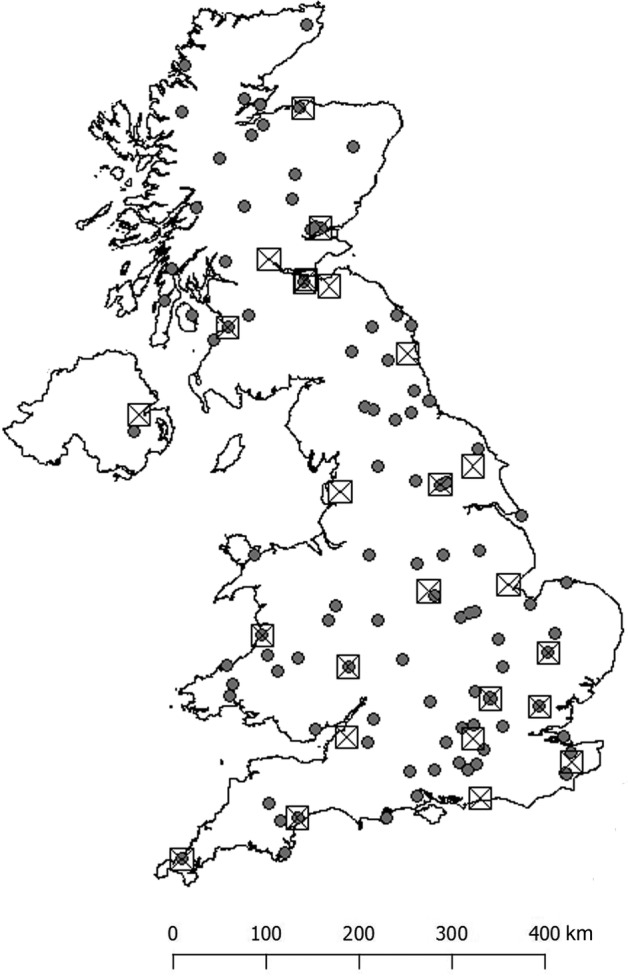
Map of the United Kingdom showing the location of the Rothamsted Insect Survey traps used in this study. Icons show light traps (filled circles) and suction traps (crossed boxes)

The Rothamsted Insect Survey operates two trap networks throughout the United Kingdom (Fig. 1) that sample aphids and moths using suction‐traps and light‐traps, respectively. Suction‐traps continuously measure the aerial density of flying aphids and other small insects, sampling at the logarithmic mean height of aphid flight (12.2 m), providing standardised daily records during the main aphid flying season (April to November) and weekly records at other times (Macaulay *et al*., 1988; Bell *et al*., 2015). The 12.2 m suction traps provide an absolute population measurement for which insect densities can be sampled more precisely than all other methods of aerial capture, operating uniformly over a wide range of conditions (Macaulay *et al*., 1988; Henderson & Southwood, 2016). The volume of air is standardised to 45 m^3^ min^−1^ and each trap in the network is calibrated annually to meet this value.

Light‐traps provide a nightly count of individual moth species from a wide range of habitats and standardised across the network by trap design, particularly the use of a clear 200 W tungsten bulbs that have a wide wavelength spectrum (400–700 nm) and the trap height at 1.2 m as described by Williams (1948) and later Fox *et al*. (2010). Each trap has a dark lid to avoid attracting higher‐flying species and is fitted with astronomical timers that shift in time as the season changes, switching the tungsten bulb on at dusk and off at dawn throughout the year since the light‐trap network's inception in 1964. Rothamsted light‐traps attract a diversity of moth species that represent a relative measure of abundance, subject to moth activity, moon phases and weather but are a consistent and representative sample of the local moth populations in flight (Taylor, 1974; Yela & Holyoak, 1997). Only macro‐moths are considered and once caught, these individuals are then identified and recorded. The attraction radii of low power light bulbs for moths have been shown to be less than 30 m (Truxa & Fiedler, 2012; Merckx *et al*., 2014) and although the tungsten bulb used here is likely to penetrate over a greater distance, in the region of 50 m, these traps still only sample the local fauna. In both networks, all target taxa are identified to species level where possible, with some difficult taxa to genus, or family level where appropriate. More information about the network can be found at https://insectsurvey.com/.

### *Data*

Data were extracted from the RIS database ‘Paul’ that holds records of moths and aphids. For each site‐year between 1969 and 2016, we calculated the total number of individuals summed across all species caught in that site‐year. These annual count data were used to estimate the overall trend in insect numbers per site‐year over time. For the suction‐traps, the total number of sites studied was 25, and these varied in time series length between 9 and 48 years, totalling 725 site‐years, representing 16 447 869 individuals. The total number of sites that contributed to the light‐trap analysis was much greater at 112. The time series varied in length between 9 and 48 years and totalled 2568 site‐years, representing 7 593 437 individuals. There was no variation in habitat for the suction‐trap network, given that they are almost exclusively based in agricultural farmland. Nonetheless, eight major habitat types are known in the light‐trap network and these are used to test whether declines vary according to habitat type.

### *Statistics*

**Figure 2:**
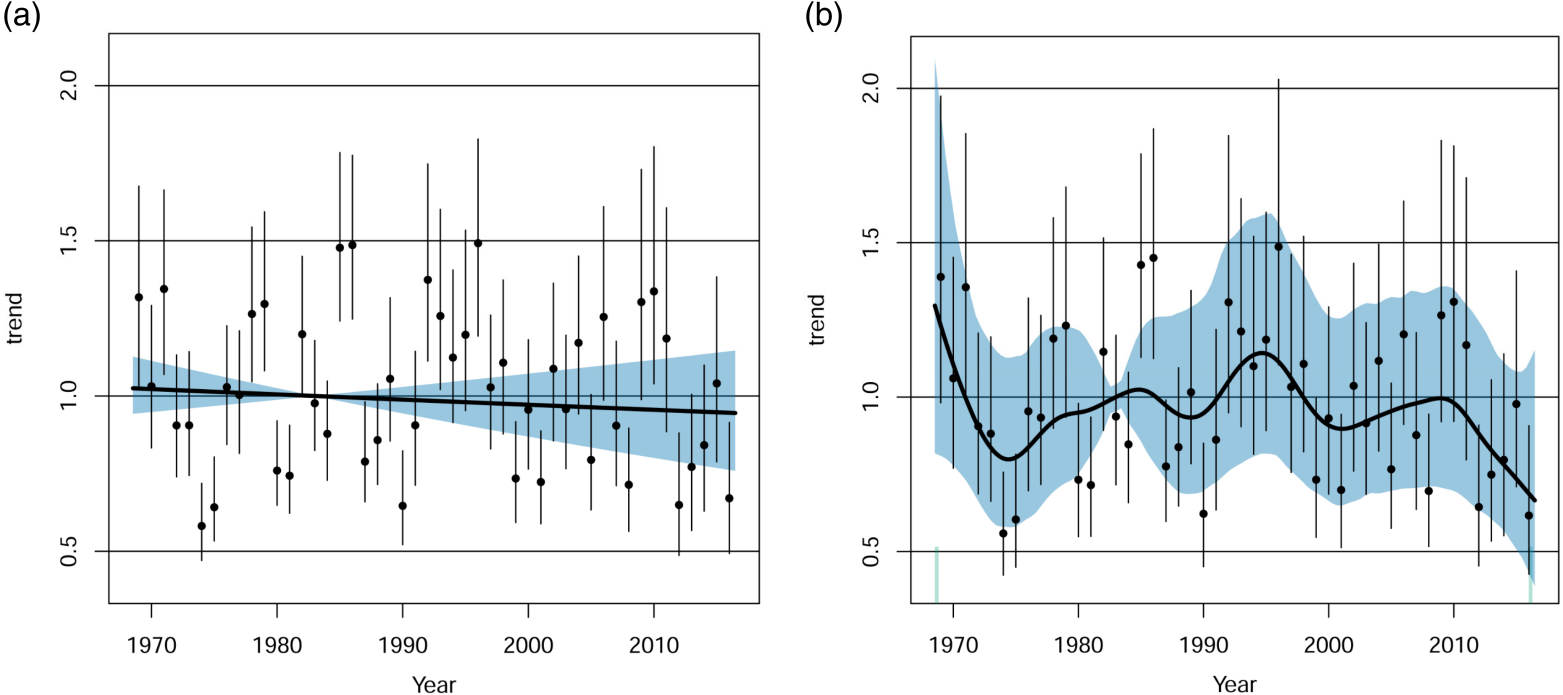
Population index for aphids with random effects (dots and whiskers), indicating yearly mean and variances, and 95% confidence intervals (blue). Two models were run (a) a log‐linear GLMM (b) a very flexible non‐linear GAMM model with d.f. fixed to *k* = 46 that follows the interannual variation over time. Neither the short‐ or long‐term trends were significant. [Color figure can be viewed at http://wileyonlinelibrary.com]

**Figure 3:**
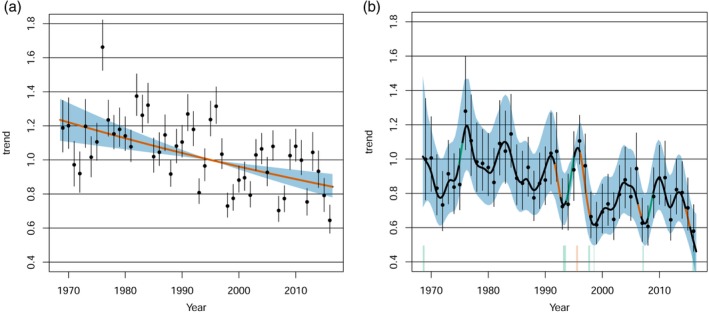
Population index for moths with random effects (dots and whiskers), indicating yearly mean and variances, and 95% confidence intervals (blue). Two models were run (a) a log‐linear GLMM (b) a very flexible non‐linear GAMM model with d.f. fixed to *k* = 46 that follows the interannual variation over time. Significantly different increasing or decreasing short term trends at the 5% level are coloured green and orange, respectively. These short‐term trends are imposed on top of the long‐term trend, which is coloured black. The shape of change (i.e. second derivatives) is indicated along the x axis, illustrating downturning (**∩** − orange bars) or upturning (∪ – green bars) curvatures in the trend. [Color figure can be viewed at http://wileyonlinelibrary.com]

All analyses were carried out in R version 3.5.0 (R Core Team, 2018). To estimate and plot annual population trends, we used the library poptrend, an extension of the mgcv library for generalised additive models and generalized additive mixed models (Knape, 2016; Wood, 2017). A log‐linear model was fitted with either a negative binomial distribution (aphids) or a quasipoisson distribution (moths) using restricted maximum likelihood (REML). The choice of distribution was based on whether a higher deviance was explained by distribution type (quasipoisson vs negative binomial). Effectively, these models were generalised linear mixed models (GLMMs) because there were no smooth components, models used exponential distributions and random effects were present (Supporting Information Appendix S1). Random effects were treated as a special case of smooths using bs = ‘re’ that computes a random coefficient for both continuous and factor variables. Simple random effects included latitude and longitude with an additional habitat term to capture the variation across light‐traps sites in different landcover types. Within the suction‐trap network, altitude did not vary widely (0–150 m), but within the light‐trap network, traps were situated from sea level to mountains (0–800 m); consequently, we included altitude as a random term within the moth analyses (Bell *et al*., 2019). For both light‐ and suction‐trap network data sets, we also fitted s(year,site,bs = ‘re’) as a random effect structure that estimated a smoother for each level of site, accounting for different trends among sites that could occur potentially as an artefact of time series length or other stochastic processes. The ptrend function also sets up temporal random effects estimates that are plotted as points with whiskers within the trend graph (see Figs. 2 and 3), to support the estimation of short‐ and long‐term trends. Further information can be found at https://github.com/jknape/poptrend. Discrete‐time autocorrelation models (corAR1) were investigated to examine whether temporal autocorrelation within the residuals of the models was present. Using mgcv's gamm routine that uses penalised quasi‐likelihood (PQL), corAR1 parameters were extracted via a call to the gamm's linear mixed‐effects model component. Weak but detectable positive autocorrelations (i.e. aphid Phi = 0.1320335; moths Phi = 0.0683) were present, although it should be noted that these models are computed using different algorithms and parameterisations than poptrend. Unfortunately, the corAR1 function cannot be implemented in poptrend ‘trend’ function. Even so, Phi values close to zero would have little effect on model outcomes.

Within the poptrend library, we implemented the ciBase function to fit the confidence intervals to the densest part of the time series, based on the number of traps running within each network. Using this approach, confidence intervals were computed from the 0.025 and 0.975 quantiles of the bootstrap distributions, based on the reference year of 1995 (light‐traps – between 1991 and 2010 the numbers of traps was >60 reaching a high of 70 in 1995) and 1983 (suction‐traps – between 1980 and 1987, 23 traps were running, for which the middle value of that series was used).

To estimate non‐linear trends, we fitted a generalised additive mixed model (GAMM) using the specification as per the log linear model but instead used generalized cross validation (GCV), the default method in poptrend (Supporting Information Appendix S1). Our aim was to fit a model that closely followed the lumps and bumps in the time series; GCV performed better than REML in this regard because REML penalises wiggliness (Wood, 2017). The number of knots, known as the basis dimension, increased to 1 minus the available total degrees of freedom for year (i.e. *k* = 46) which GCV appropriately exploited. A unique property of poptrend is an explicit test for significance in the short‐term trend estimates, nested in the overall smoothed trend. If detected, these short‐term trends are depicted in orange for decline and green for an increase, as seen above in Fig. 3. These short‐term trends are imposed on top of the long‐term trend, coloured black. All trends are relative measures that are standardised against the total predicted count in the first year, the reference year.

A log‐linear sensitivity analysis was run to examine whether a small number of dominant species were driving overall trends and thus saturating the models. For aphids, three species accounted for 51% of the total number of individuals caught and were ever‐present (*Rhopalosiphum padi*, *R. oxyacanthae*, and *Sitobion avenae*). These species are all major pests of cereal crops in the United Kingdom. The combined annual total count of these three species was compared against the total count of the remaining subset of species and log‐linear trends estimated (Supporting Information Fig. S1; Table S1). A different approach was taken for the moths due to the fact that dominant species tended to turnover much more frequently than for aphids. The top 10 moth species per decade over the whole of the time series were combined, amounting to 19 common species that represented 29% of the total moth count (Supporting Information Table S2). These were combined and compared against the remaining species annual total count subset, using log‐linear trends as before (Supporting Information Fig. S2; Table S1). The rate of change and the confidence intervals were of principal interest.

To estimate non‐linear habitat differences between light‐trap catches over time, we used GAMMs within mgcv to explore the interaction between the year smooth term and habitat as a factor variable (i.e. s(Year, bs = ‘ts’, *k* = 46, by = PrimaryLandCover)). This yielded trends for each habitat over time, centred at zero with a common mean (i.e. intercept). Importantly, we also cast PrimaryLandCover, the description for habitat within the data set, as a parametric term (Supporting Information Appendix S1). This allowed means that naturally differed between habitats to be considered. These differences are of limited use because these parametric terms are tested against the reference habitat, coastal but are reported separately for completeness. The models used the GCV method as before. Model checking using mgcv diagnostics was performed routinely, particularly Q‐Q probability plots of standardised residuals against covariates, supporting the chosen distribution, model fit and the degree of smoothing.

For all models, a smoother for each level of site, accounting for different trends among sites using s(year,site,bs = ‘re’) random effect structure, was estimated. Generally, this produced stable models for all but the non‐linear moth model that was severely compromised (Supporting Information Figs. S3, S4; Table S3). A more stable model with a simple s(site,bs = ‘re’) random effect structure is included within the main body of this article. We refer to both models in our results.

Independently of the GLMMs and GAMMs, we used multivariate spline cross correlograms to estimate the spatial dependence of the annual counts as a continuous function of distance using the R library ncf (Bjørnstad, 2019). These correlograms elucidate the spatial scale of insect population change using the local covariance function, the spatial extent and the correlation length parameters to estimate the strength of spatial synchrony. The multivariate spline generally infers whether spatial synchrony is local or widespread in space and often shows synchrony declining with distance to a point of randomness, but may also show chaotic local dynamics. Definitions used and examples can be found in the study by Bjørnstad *et al*. (1999); a moth example over very large spatial scales can be seen in the study by Bell *et al*. (2012a). More details on the method can be found in the studies by Bjørnstad *et al*. (1999) and Bjørnstad & Falck (2001).

## Results

**Figure 4:**
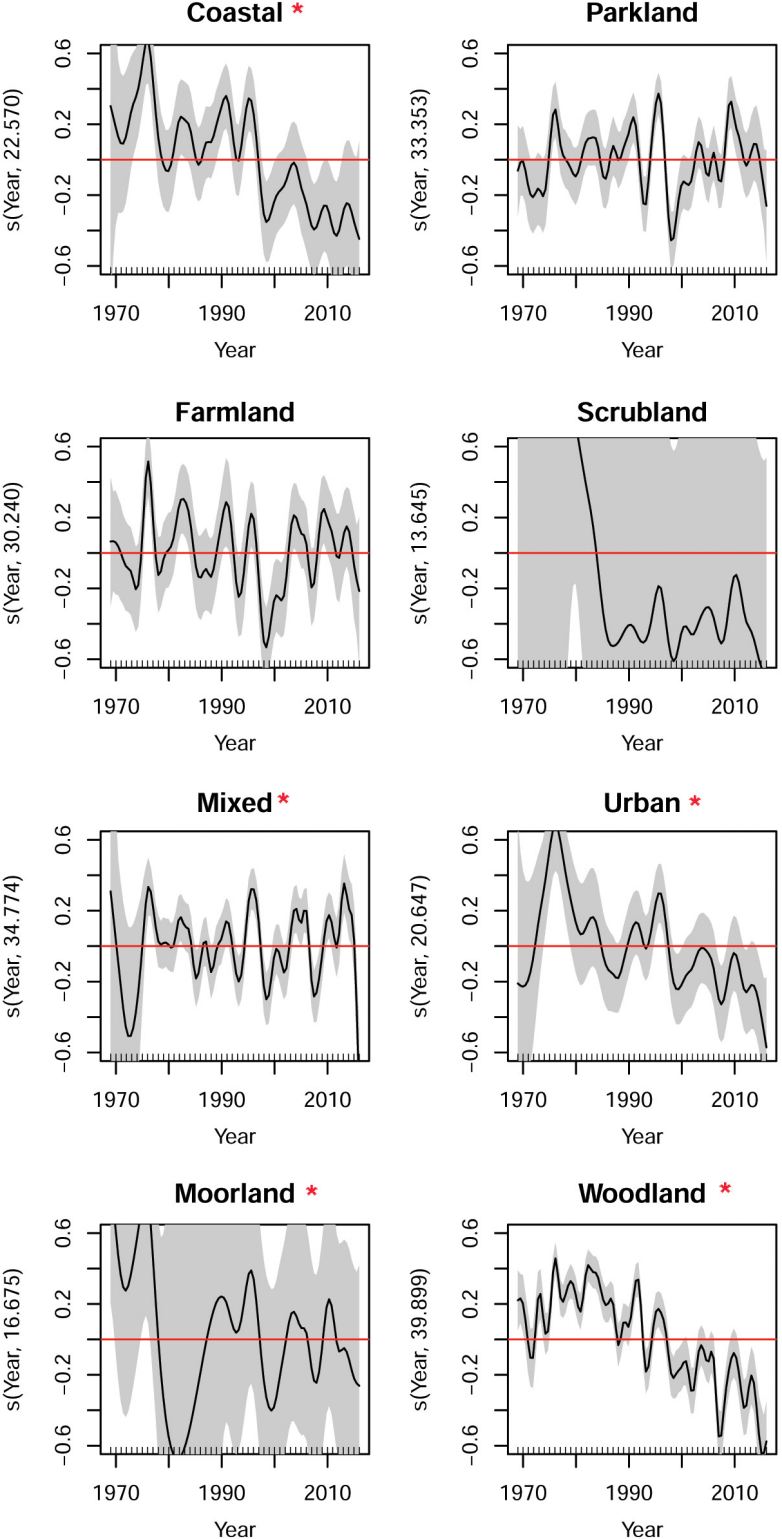
Effect of habitat on non‐linear moth declines from the model featured in Fig. 3b. The estimated smoothed terms on the *y* axis are a transformed function of habitat which on the axis is centred on zero (red line). For each habitat plot, a Wald zero‐effect test indicates if the smoother is equal to zero. Significant *P*‐values show that smooths have significantly departed from zero. A formal test is provided in Table 1 and significant trends are asterisked * accordingly. In only coastal, urban and woodland could the trends be described as significantly declining. Both mixed and moorland habitats fluctuate widely, and we are cautious about the significance attached to those trends. [Color figure can be viewed at http://wileyonlinelibrary.com]

The estimated long‐term linear percentage change in the trend (with 95% confidence intervals) for aphids was −7.6% (−32%, 22%) and the estimated long‐term non‐linear percentage change was −44% (−71%, 0.5%), caused by an initial decline during the 1970s and falling annual totals after 2010. In both cases, these long‐term change estimates were non‐significant and no significant short‐term trends were identified either (Fig. 2a,b). The estimated long‐term linear percentage change in the trend for moths was −31% (−42%, −18%) and the estimated long‐term non‐linear percentage change was −48% (−62%, −29%), with significant sharp declines in the late 1970s, the early and late 1990s, late 2000s and mid 2010s (Fig. 3a,b). It is notable that moth populations went through periods of recovery in the late 1970s, mid 1990s and a short period before and including 2010. It should be noted that the s(year,site,bs = ‘re’) random effect structure for the non‐linear moth model was a highly significant term (t111 = 96 633 628, *P* = <0.0001), suggesting that there were different trends among sites. Yet, the more conservative rate of change of −43% produced exceptionally wide confidence intervals (−99%, 4367%) (Supporting Information Fig. S3). A sensitivity analysis revealed that the log‐linear rates of change in moths were not driven by dominant species: instead ‘uncommon’ and ‘dominant’ species reported very similar rates of log‐linear change (Supporting Information Fig. S2). In aphids, the top three most abundant species (which accounted for 51% of individuals) declined significantly in abundance by −42% while the remaining subset showed a non‐significant increase of 23% (Supporting Information Fig. S1). The effect of habitat on moth populations yielded variable results (Figs. 4 and Supporting Information Fig. S4). Just over half the types reported significant habitat effects and these were for coastal, urban, moorland, mixed and woodland habitats (Table 1; Fig. 4) and none of these smoothers were significant for the model which included s(year,site,bs = “re”) random effects structure (Supporting Information Table S3). Notably, a decline was not detected in farmland habitats in either non‐linear model (Table 1; Supporting Information Table S3).

**Table 1:** Results from non‐linear GAMM of moth trends presented in Figs. 3b and 4 that show test results for the interaction between year smooth term and habitat as a factor variable (Supporting Information Appendix S1). The parametric coefficients for habitat levels report differences relative to the reference habitat, coastal and are of limited use but included here for completeness. The lower the effective degrees of freedom (edf) of the smoother suggests a more linear trend. The significance of some of these smoothers term should be viewed cautiously in the non‐linear model because the trend fluctuates widely around the zero mean (red line) in much the same way as parkland (Fig. 4), a non‐significant term. further, when a year‐by‐site random effects structure was used, the presumably lack of data in each habitat class contributed to the non‐significant smoothers reported (Table S3).

**A. Parametric coefficients**	**Estimate**	**Std. Error**	***t*‐value**	***P*‐value**
(Intercept)	7.356	0.327	22.486	<0.0001
as.factor:farmland	0.330	0.396	0.833	0.405
as.factor:mixed	0.419	0.393	1.067	0.286
as.factor:moorland	0.073	0.558	0.130	0.897
as.factor:parkland	0.129	0.367	0.352	0.725
as.factor:scrubland	0.523	0.790	0.662	0.508
as.factor:urban	−0.316	0.389	−0.812	0.417
as.factor:woodland	0.941	0.380	2.477	0.013
**B. Smooth terms**	**edf**	**Ref.df**	***F*‐value**	***P*‐value**
s(Year):coastal	22.570	45	53.698	<0.0001
s(Year):farmland	30.240	45	19.378	0.062
s(Year):mixed	34.774	45	14.711	0.001
s(Year):moorland	16.675	35	59.356	<0.001
s(Year):parkland	33.353	45	11.547	0.200
s(Year):scrubland	13.645	35	7.935	0.059
s(Year):urban	20.647	45	89.894	0.001
s(Year):woodland	39.899	45	591.728	<0.0001
s(Site)	102.659	104	105.114	< 0.0001
s(Latitude)	0.000	1	0.000	0.813
s(Longitude)	0.002	1	0.006	0.737
s(Altitude)	0.002	1	0.010	0.669

The number of sites for each habitat type is as follows: Coastal = 7; Farmland = 15; Mixed = 16; Moorland = 6; Parkland = 27; Scrubland = 4; Urban = 17; Woodland = 20.

**Figure 5:**
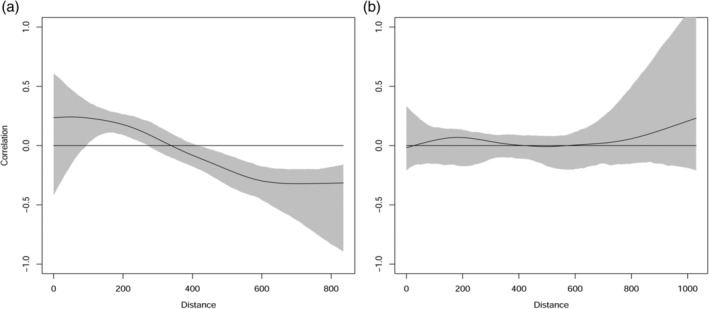
Multivariate spline correlograms with bootstrapped 95% confidence intervals of the annual catch of across Great Britain (a) Aphids. The *y* intercept indicates the local covariance function (0.23), the *x*, the spatial extent (0–800 km) and the *x* intercept is an estimate of the correlation length (338 km) (b) Moths. The *y* intercept indicates the local covariance function (0), the *x*, the spatial extent (0–800 km) and the *x* intercept is an estimate of the correlation length (0 km).

Multivariate spline cross correlograms for aphids showed that, after a degree of high variation locally, the spatial extent of the autocorrelation decayed to a distance of 338 km at which point it was random (Fig. 5a). For moths, the local covariance function was not significantly different from zero (Fig. 5b), in other words, there was no spatial autocorrelation over the entire correlation length.

## Discussion

Our research showed that the annual count of moths in Great Britain is in significant decline, estimated to be −31% according to the long‐term linear trend and in precise agreement with Conrad *et al*. (2004, 2006) and close agreement with Fox *et al*. (2013). A new insight has been gained from the non‐linear models produced in this article, highlighting periods of significant decline and recovery in all decades except the 1980s. The non‐linear trend supports the findings of MacGregor *et al*. (2019) who demonstrated that moth biomass increased from 1967 to 1982 and started to decline only after the early 1980s. It is beyond the scope of this article to unpick the causes of these short‐term trends, but it would seem unlikely that climate alone is responsible. After all, the 10 warmest years since 1884 have all occurred since 2002 and, between 1981 and 2010, the temperature was 0.9°C warmer compared to 1961–1990, with much less severe winters (Kendon *et al*., 2019). These conditions should broadly favour moths and yet the analysis here showed a decline. Fox *et al*. (2013) showed that trends varied among regions, citing southern Britain where moth decline was the steepest (−40%), implicating land use changes more than climate. Indeed, Dieker *et al*. (2011) inferred that this was likely the case for alpine moths, where land‐use changes exceeded the impact of climate change; it has also been argued for agricultural invertebrates too (Ewald *et al*., 2015). Against this, it must be noted that we found no evidence of moth declines in agricultural habitats, a similar result to aphid trends in that habitat. This study appears to contrast with Seibold *et al*. (2019) who found that invertebrates in grassland habitats were declining at a faster rate than woodland. The two studies are not strictly comparable; in our 47‐year study, grassland is found within more general farmland and parkland habitat types, compared to a decade's worth of monitoring using discrete 50 m habitat plots in the Seibold study. Importantly, we have demonstrated that shorter time periods can show great variability that linear models alone may not capture.

Anecdotally, butterflies and moths have been in a period of decline for more than 150 years in Great Britain, casually linked by naturalists (Ford, 1945; Bretherton, 1951; Ford, 1955) and scientists (Taylor, 1974; Conrad *et al*., 2004, 2006; Woiwod & Gould, 2008; Fox *et al*., 2014) to changes in land use. This assertion is justified, given the dramatic change in agriculture in post‐war Britain, which now represents 72% of the land area (Defra, 2019). The decline in moths may have been most severely felt when, during the 1950s, Britain went through an agricultural revolution that saw widespread mechanisation and routine organochlorine insecticide and inorganic fertiliser use (Robinson & Sutherland, 2002). These developments led to the demise of fallow in rotational agriculture and a dramatic increase in field size. Booming agriculture saw the reduction in both extensive low‐input cultivation and non‐cropped habitats and it is these changes in particular that were implicated in moth population declines cited above. Nevertheless, whilst agricultural intensification has been given special mention by these and other authors, it alone does not explain fully why moths are declining in semi‐natural environments, where other pressures, such as light pollution, urbanisation and disturbance may also play a part, particularly in southern Britain. The problem of implicating these and other drivers in a formal statistical test is limited because of the absence of high‐quality long‐term land use and environmental data. What we are able to observe, in lieu of land use and environmental data, is that there are clear differences in the rate and direction of change between habitats, which may also explain the lack of spatial synchrony that suggests high variation locally between sites. We found that moths recorded in coastal, urban, woodland, mixed and moorland habitats were significantly different from zero, but only coastal, urban and woodland habitats showed declines over time (Table 1). Surprisingly, agricultural habitats do not report a decline, but this may be because moths in this habitat have already lost those species at greatest extinction risk during the period of dramatic landscape change during the 1950s. This is an area of current interest, which requires manipulative experiments to unravel how moth populations in agricultural habitats can be understood.

Conversely, aphids do not share a decline phase in the same way as moths, but instead are broadly stable between 1969 and 2016 (−7.6%) according to the linear trend. Our analysis using annual counts with a negative binomial distribution is a more comprehensive analysis for detecting the rate of change than Bell *et al*.'s (2015) previous assessment. In that study, Bell *et al*. (2015) showed a moderate non‐linear significant increase overall using log annual counts in a Gaussian model framework. Clearly, aphids display a great deal of interannual volatility and transforming the response is not without its merits, especially considering the wide confidence intervals produced from linear trend modelling with a negative binomial distribution in this article (i.e. −32%, 22%). To compare and provide an independent perspective, the evidence from the field is that summer aphid populations in cereals have declined over 40 years (Ewald *et al*., 2015), in agreement with our current study that showed a significant decline in three major cereal pests. It is perhaps surprising that no significant short‐term trends were highlighted in the non‐linear model, given their status as a primary target for insecticides and the propensity for aphid populations to outbreak over wide areas. But it is likely that the exponential growth of aphids, particularly those aphids associated with cereals and beans that reach extreme numbers during peak infestation, would have been averaged out in this analysis by other species that do not reach outbreaking levels (Bell *et al*., 2015). What remains consistent between the few studies that have examined aphids over long periods of time, is that whilst a linear trend is informative for broad statements about trends, aphids are fundamentally volatile within‐ and between‐years. Extreme variance suggests that we should think of aphids as a ‘special case’ for which the concept of outbreak with interannual variation is perhaps more appropriate paradigm. That said, aphids are remarkable for their migration which produced broadly spatially synchronous regional trends in this study, albeit with a high degree of local variation.

Implicating the causes of a broadly stable trend in aphids does raise similar issues to that discussed for moths. For example, although the Department for Environment, Food and Rural Affairs (Defra) do provide a very coarse regional measure every few years (Defra, 2019), annual records of insecticide use are absent at the spatial (i.e. 5 km scale) or compound (i.e. aphicides) resolution needed. The CEH Land Cover maps have now been updated to include pesticide usage (Jarvis *et al*., 2019) and show potential. But, at present, the series is limited to 2012–2016, which precludes its use in this study. Ewald *et al*. (2015) has shown that insecticide usage has driven down some of the beneficial insect groups as well as aphids. As foliar applications of pyrethroids and seed‐treated clothianidin were applied in nearly all arable fields at least once per annum in recent years, it is perhaps unsurprising that the three cereal pest species studied separately here have declined. Although, with the withdrawal of neonicotinoid treated seed mandated for all crops sown in autumn 2019, including non‐flowering ones, it is expected that the annual count of aphids should rise if no other effective check on populations is found. Other drivers of change, such as host plants, have been modelled for migrating aphids (Bell *et al*., 2012b). These data can, at best, only provide information on how widespread hosts are, not necessarily how locally abundant they may be, which is needed. We know from Bell *et al*. (2015) and Ewald *et al*. (2015) that weather and climate determine the long‐term trends in the number of aphids, but even here increasing accumulated degree days has shown only to produce small, positive changes in annual counts over decades with substantial unaccounted variation. Clearly, to untangle these drivers will require finely tuned individual species models.

We are confident of both the sign and rate of change reported here exceed the requirements given by Wauchope *et al*. (2019), in that we present long‐term, standardised, regularly sampled data and report confidence intervals around our modelled trends. We are also confident that the reported declines in moths are not driven by changes in the commonest species as grouping ‘dominant’ and ‘uncommon’ species separately found no significant difference in the sign or rate of change. In aphids however, there is evidence that the three most common agricultural pest species (*R. padi*, *R. oxyacanthae* and *S. avenae*) are showing significant long‐term population declines, possibly because of control measures. This was not true for less common aphid species, which had a stable population trend over time. The implications for insect declines research is profound. The decline in insects is being felt across trophic levels and one of the major concerns is the potential impact on insectivorous birds. Aphids and moths both form important components of the bird diets (Wilson *et al*., 1999; Holland *et al*., 2006; Holland *et al*., 2012). Møller (2019) showed that Danish barn swallows, house martins, and sand martins decreased significantly over a 20‐year period, linking the decline to the availability of insects. Bowler *et al*. (2019) showed that declines in insectivorous birds was most strongly felt in agricultural habitats, particularly grasslands, that caused bird communities to be dominated by diet generalists. On the North American continent, similar patterns are apparent (Fitzgerald *et al*., 2014; Michel *et al*., 2016). In Canada, chimney swifts (*Chaetura pelagica*) have been linked to insect community change over a 48‐year period using dietary reconstruction methods. The study showed that chimney swifts are in decline due to widespread use of the pesticide DDT in the 1960s, which correlated with a significant change in prey and therefore nutrients (Nocera *et al*., 2012). Further, an isotope analysis of Eastern whip‐poor‐will (*Caprimulgus vociferus*) over ~130 years found some evidence of a shift in diet away from higher trophic level insect prey (English *et al*., 2018). Clearly, more research is needed to examine species diversity and community composition, concentrating at the field level over long periods of time and measuring covariates like weather, insecticide usage, landscape and habitat change. Insect decline science is limited by funding, but the arguments are compelling and include known‐knowns, such as the link between aerial insect numbers and insectivorous birds. Other aspects are much less well understood, such as the displacement of insect numbers and the consequences for trophic mismatching, and the homogenisation of insect communities that increasingly include diet generalists and common eurytopic species.

## Supporting information


**Appendix S1.** Supporting information

**Figure S1.** Population index for (a) the top three most common aphids and (b) the remaining subset of aphids, showing random effects (dots and whiskers) indicating yearly mean and 95% confidence intervals (blue).
**Figure S2.** Population index for (a) the top 19 most common moths and (b) the remaining subset of moths, showing random effects (dots and whiskers) indicating yearly mean and 95% confidence intervals (blue).
**Figure S3.** Output from non‐linear model including year‐by‐site random effect. Population index for moths, showing year random effects (dots and whiskers) indicating yearly mean and 95% confidence intervals (blue). Estimated percent change from Year = 1969 to 2016: −43% (−99%, 4367%).
**Figure S4.** Effect of habitat on non‐linear moth population change from model including a year‐by‐site random effect. The estimated smoothed terms on the *y* axis are a transformed function of habitat which on the axis is centred on zero (red line).
**Table S1.** Model summaries for aphid and moth log‐linear GAMMs, run separately for the most common species and the remaining subset of each group.
**Table S2.** The top ten most numerous moth species caught in each decade of the study, amounting to 19 species.
**Table S3.** Model summaries for non‐linear moth population trend models including a year‐by‐site random effect, both without and with an interaction term for habitat.
