# Supplementary material for: Are insects declining and at what rate? An analysis of standardised, systematic catches of aphid and moth abundances across Great Britain
Source: Insect Conserv Divers. 2020 Mar 4;13(2):115–26. doi: 10.1111/icad.12412 (PMC7079554; doi:10.1111/icad.12412)
Supplement: Supplementary file 1 — Appendix S1. Supporting information [file ICAD-13-115-s001.docx]

**Appendix S1** Structure of poptrend models used

# required

library(poptrend)

library (mgcv)

# Aphids

*# Log-linear GLMM models*

A <- ptrend (Count ~ trend(Year, tempRE = TRUE, type = "loglinear") + s(Year,Site,bs="re") + s(Latitude, bs = "re") + s(Longitude, bs = "re"), family = nb(5.79231), data = aphids, gamModel=TRUE)

*# Non- linear GAMM models*

Awiggle <- ptrend (Count ~ trend(Year, k=46, tempRE = TRUE, type = "smooth") + s(Year,Site,bs="re") + s(Latitude, bs = "re") + s(Longitude, bs = "re"), family = negbin(theta = 5.792535, link = "log"), method = "GCV.Cp", data = aphids, gamModel=TRUE)

# Moths

*# Log-linear GLMM models*

M <- ptrend (Count ~ trend(Year, tempRE = TRUE, type = "loglinear") + s(Year,Site,bs="re") + s(Latitude, bs = "re") + s(Longitude, bs = "re") + s(Altitude, bs = "re") + s(PrimaryLandCover, bs = "re"), family = quasipoisson,  method = "REML", data = moths, gamModel=TRUE)

*# Non- linear GAMM models*

# Mwiggle.site.trend.re has s(Year,Site,bs="re") which produces a model with extremely high uncertainties (see Appendix S2)

Mwiggle.site.trend.re <- ptrend (Count ~ trend(Year, k=46, tempRE = TRUE, type = "smooth") + s(Year,Site,bs="re") + s(Latitude, bs = "re") + s(Longitude, bs = "re") + s(Altitude, bs = "re") + s(PrimaryLandCover, bs = "re"), family = quasipoisson, method = "GCV.Cp", data = moths, gamModel=TRUE)

# Does not have complex random effects of year and site combinations s(Site,bs="re")

# trend much more stable and used in main body of paper

Mwiggle <- ptrend (Count ~ trend(Year, k=46, tempRE = TRUE, type = "smooth") + s(Site,bs="re") + s(Latitude, bs = "re") + s(Longitude, bs = "re") + s(Altitude, bs = "re") + s(PrimaryLandCover, bs = "re"), family = quasipoisson, method = "GCV.Cp", data = moths, gamModel=TRUE)

*# Non- linear habitat GAMM models*

Mwiggle.hab <- gam(Count ~ s(Year, bs="ts", k=46, by = PrimaryLandCover) + as.factor(PrimaryLandCover) + s(Site, bs="re") + s(Latitude, bs = "re") + s(Longitude, bs = "re") + s(Altitude, bs = "re"), method = "GCV.Cp", family = quasipoisson, data = moths)
