# Supplementary material for: Are insects declining and at what rate? An analysis of standardised, systematic catches of aphid and moth abundances across Great Britain
Source: Insect Conserv Divers. 2020 Mar 4;13(2):115–26. doi: 10.1111/icad.12412 (PMC7079554; doi:10.1111/icad.12412)
Supplement: Supplementary file 2 — Figure S1. Population index for (a) the top three most common aphids and (b) the remaining subset of aphids, showing random effects (dots and whiskers) indicating yearly mean and 95% confidence intervals (blue). Figure S2. Population index for (a) the top 19 most common moths and (b) the remaining subset of moths, showing random effects (dots and whiskers) indicating yearly mean and 95% confidence intervals (blue). Figure S3. Output from non‐linear model including year‐by‐site random effect. Population index for moths, showing year random effects (dots and whiskers) indicating yearly mean and 95% confidence intervals (blue). Estimated percent change from Year = 1969 to 2016: −43% (−99%, 4367%). Figure S4. Effect of habitat on non‐linear moth population change from model including a year‐by‐site random effect. The estimated smoothed terms on the y axis are a transformed function of habitat which on the axis is centred on zero (red line). Table S1. Model summaries for aphid and moth log‐linear GAMMs, run separately for the most common species and the remaining subset of each group. Table S2. The top ten most numerous moth species caught in each decade of the study, amounting to 19 species. Table S3. Model summaries for non‐linear moth population trend models including a year‐by‐site random effect, both without and with an interaction term for habitat. [file ICAD-13-115-s002.docx]

**Supporting information**

**Fig. S1** Population index for (a) the top three most common aphids and (b) the remaining subset of aphids, showing random effects (dots and whiskers) indicating yearly mean and 95% confidence intervals (blue).

**Fig. S2** Population index for (a) the top 19 most common moths and (b) the remaining subset of moths, showing random effects (dots and whiskers) indicating yearly mean and 95% confidence intervals (blue).

**Table S1** Model summaries for aphid and moth log-linear GAMMs, run separately for the most common species and the remaining subset of each group.

**Table S2** The top ten most numerous moth species caught in each decade of the study, amounting to 19 species.

To ensure that the overall trends in aphids and moths were not biased by the trends of the most common species, we re-ran the models on a subset of the data containing only the most common species and compared these to models run on the remaining data. As in the main models, counts of individual species were summed so that the response variable consisted of one value per site-year. Log-linear models were run in the poptrend package in R following using the following R code:

# required

library(poptrend)

library(mgcv)

# Aphids

A <- ptrend (Count ~ trend(Year, tempRE = TRUE, type = "loglinear") + s(Year,Site,bs="re") + s(Latitude, bs = "re") + s(Longitude, bs = "re"), family = nb(5.79231), data = aphids, gamModel=TRUE)

# Moths

M <- ptrend (Count ~ trend(Year, tempRE = TRUE, type = "loglinear") + s(Year,Site,bs="re") + s(Latitude, bs = "re") + s(Longitude, bs = "re") + s(Altitude, bs = "re") + s(PrimaryLandCover, bs = "re"), family = quasipoisson,  method = "REML", data = moths, gamModel=TRUE)

# Aphids

For aphids, we identified the 3 species that were the most numerous across the time series as a whole, accounting for 51% of individuals recorded. These species were *Rhopalosiphum padi* (Bird cherry-oat aphid), *Rhopalosiphum oxyacanthae* (Apple-grass aphid) and *Sitobion avenae* (English grain aphid).


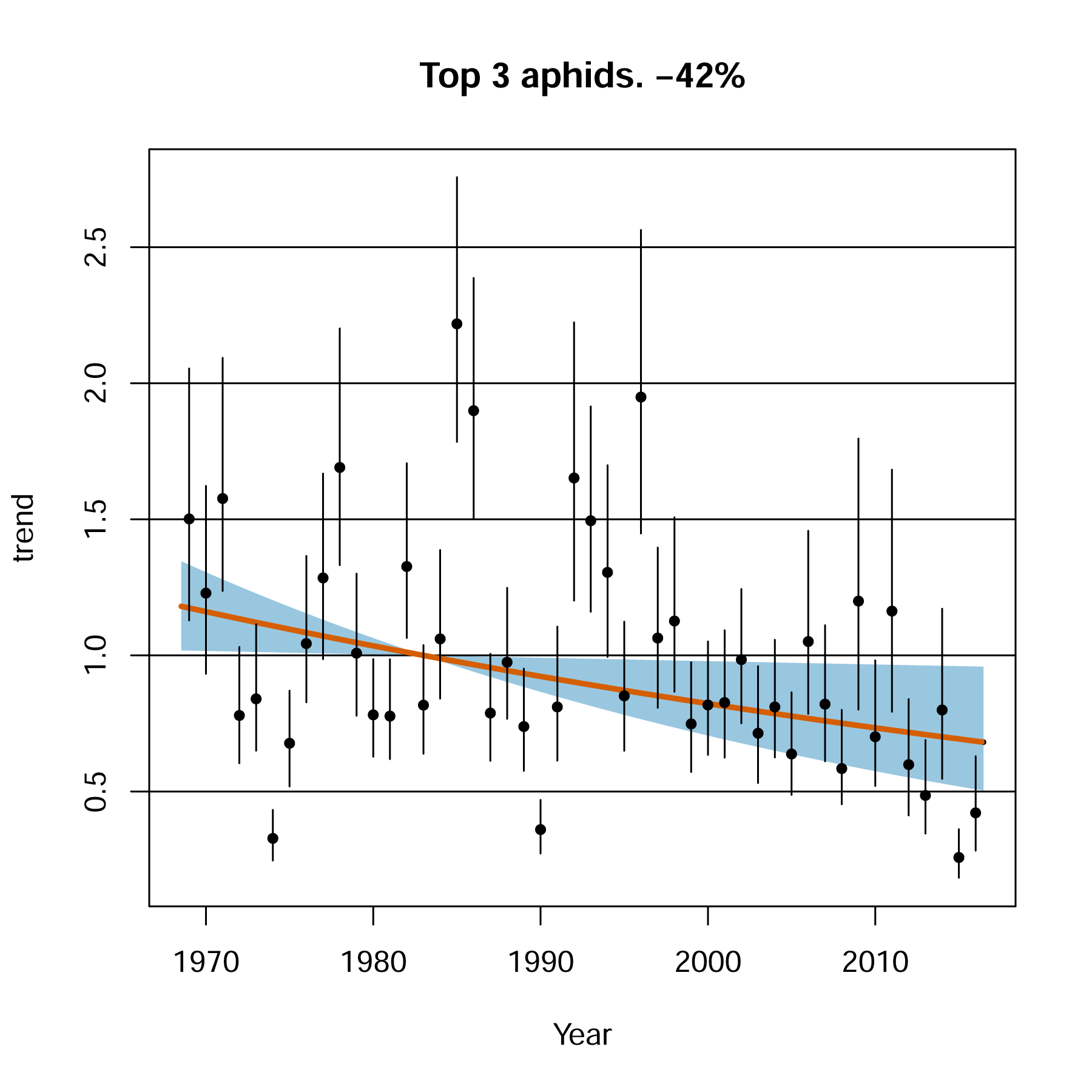


Fig. S1 (a) Population index for the top three most common aphids, showing random effects (dots and whiskers) indicating yearly mean and 95% confidence intervals (blue). There is a significant decline of -42% (95% CI -62%, -6.5%).


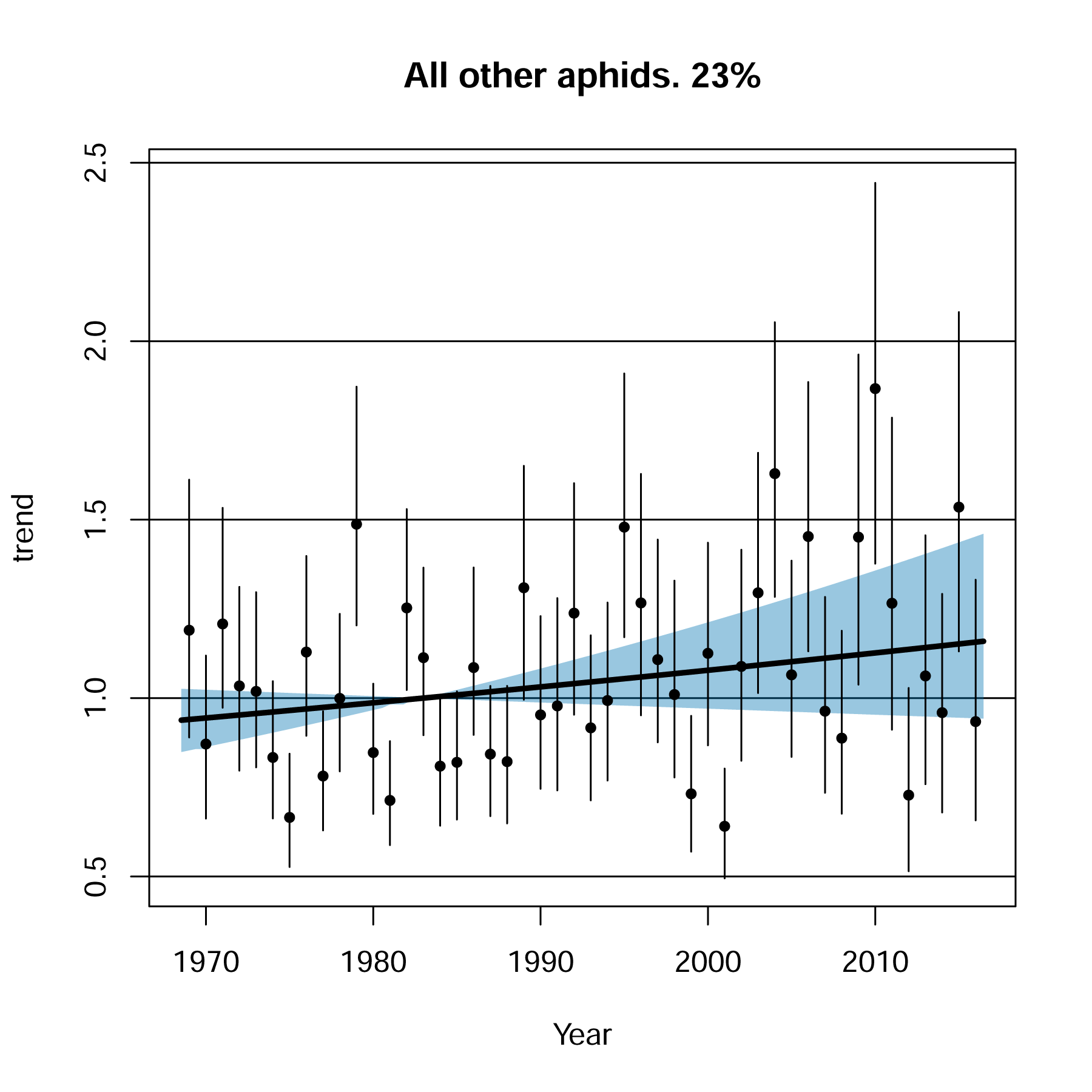


Fig. S1 (b) Population index for all aphids excluding the the top three most common, showing random effects (dots and whiskers) indicating yearly mean and 95% confidence intervals (blue). There is a non-significant increase of 23% (95% CI -7.8%, -70%).

# Moths

For moths, we identified the ten most numerous species in each decade which amounted to 19 species. These 19 species accounted for 29% of individuals recorded (Table S1)


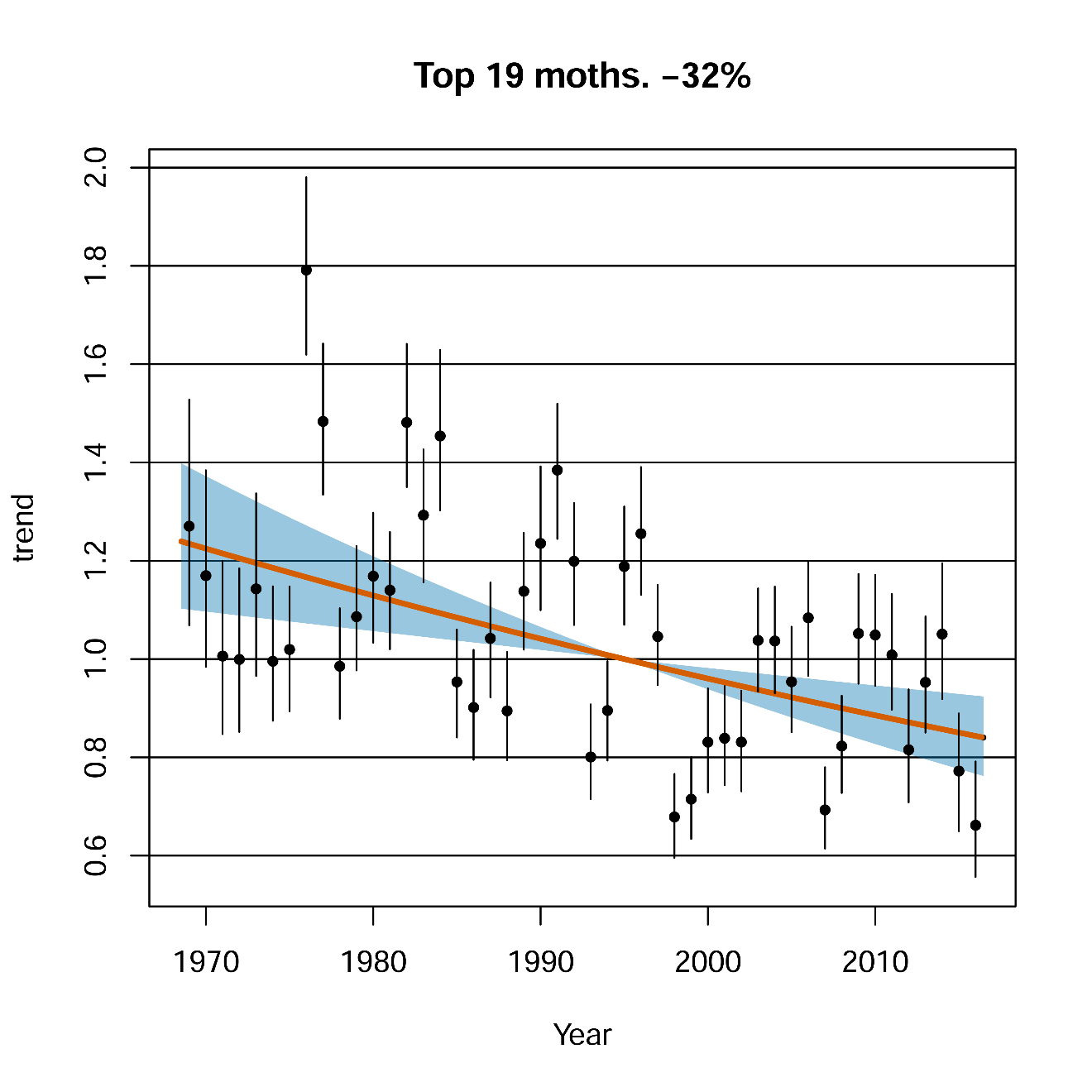


Fig. S2 (a) Population index for top 19 most common moths, showing random effects (dots and whiskers) indicating yearly mean and 95% confidence intervals (blue). There is a significant decline of -32%% (95% CI -45%, -16%).


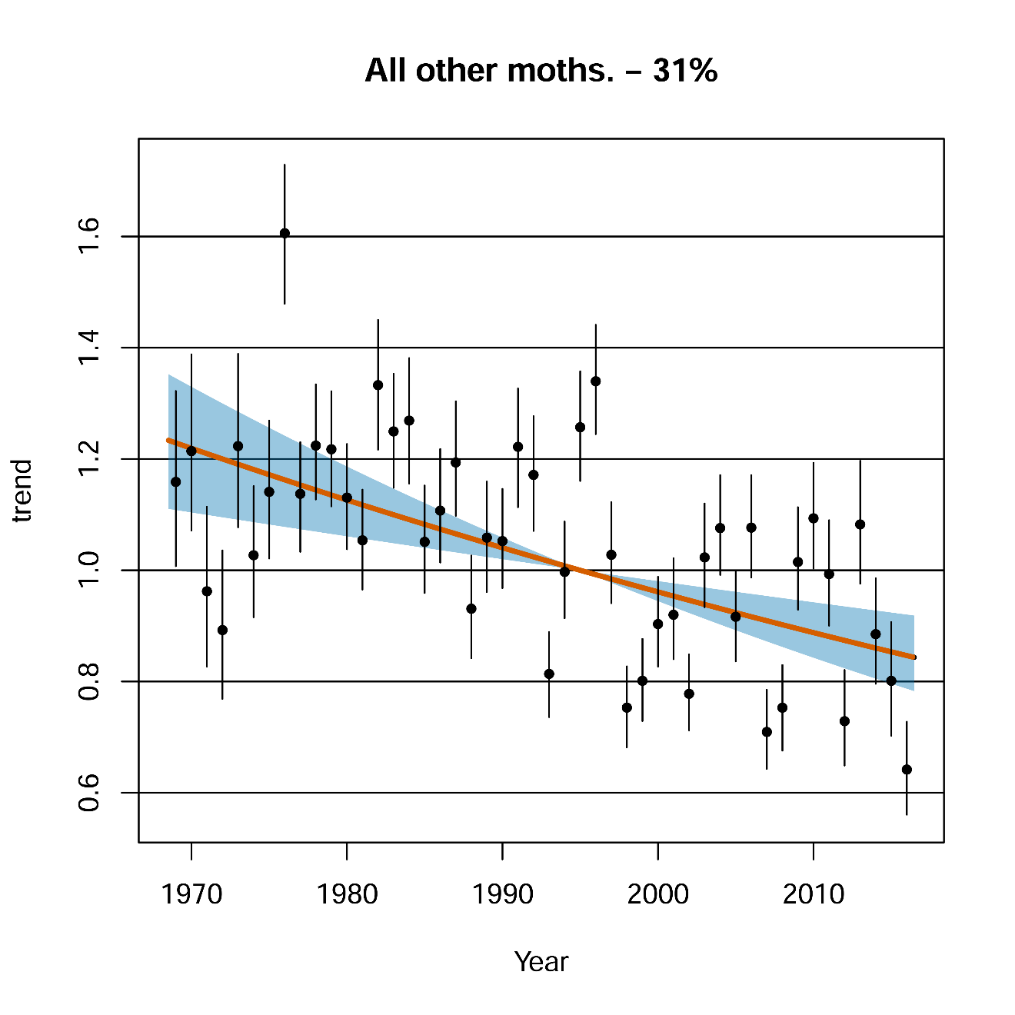


Fig. S2 (b) Population index for all moths excluding the top three most common, showing random effects (dots and whiskers) indicating yearly mean and 95% confidence intervals (blue). There is a non-significant increase of 23% (95% CI -7.8%, -70%).

Table S1. Model summaries for aphid and moth log-linear GAMMs, run separately for the most common species and the remaining subset of each group.

| Response | A. parametric coefficients | Estimate | Std. Error | t-value | p-value |
| --- | --- | --- | --- | --- | --- |
| Most common aphids abundance | (Intercept) | 26.78 | 9.967 | 2.6869 | 0.0072 |
|  | Year | -0.0115 | 0.0049 | -2.3289 | 0.0199 |
|  | B. smooth terms | edf | Ref.df | F-value | p-value |
|  | s(Latitude) | 0.8892 | 1 | 3279.967 | 0.0027 |
|  | s(Longitude) | 0.0002 | 1 | 0 | 0.8304 |
|  | s(Year,Site) | 21.55 | 24 | 476.0991 | < 0.0001 |
|  | s(Year__Fac) | 42.536 | 46 | 695.2581 | < 0.0001 |
|  |  |  |  |  |  |
| All other aphids abundance | (Intercept) | 0.6801 | 6.5872 | 0.1032 | 0.9178 |
|  | Year | 0.0044 | 0.0033 | 1.3346 | 0.182 |
|  | B. smooth terms | edf | Ref.df | F-value | p-value |
|  | s(Latitude) | 0 | 1 | 0 | 0.8692 |
|  | s(Longitude) | 0.913 | 1 | 4298.723 | 0.0007 |
|  | s(Year,Site) | 21.6322 | 24 | 385.3623 | < 0.0001 |
|  | s(Year__Fac) | 36.4287 | 46 | 202.3185 | < 0.0001 |
|  |  |  |  |  |  |
| Most common moths abundance | (Intercept) | 22.5558 | 4.5114 | 4.9997 | < 0.0001 |
|  | Year | -0.0081 | 0.0023 | -3.5824 | 0.0003 |
|  | B. smooth terms | edf | Ref.df | F-value | p-value |
|  | s(Year,Site) | 101.6959 | 110 | 1430.617 | < 0.0001 |
|  | s(Latitude) | 0.0001 | 1 | 0.0009 | 0.0745 |
|  | s(Longitude) | 0.0003 | 1 | 0.0007 | 0.5153 |
|  | s(Altitude) | 0.0025 | 1 | 0.0368 | 0.4107 |
|  | s(PrimaryLandCover) | 5.1223 | 7 | 37049.81 | 0.0008 |
|  | s(Year__Fac) | 42.4071 | 46 | 37.2119 | < 0.0001 |
|  |  |  |  |  |  |
| All other moths abundance | (Intercept) | 23.0636 | 3.7368 | 6.1721 | < 0.0001 |
|  | Year | -0.0079 | 0.0019 | -4.231 | < 0.0001 |
|  | B. smooth terms | edf | Ref.df | F-value | p-value |
|  | s(Year,Site) | 102.3561 | 110 | 11274.4 | < 0.0001 |
|  | s(Latitude) | 0 | 1 | 0 | 0.2837 |
|  | s(Longitude) | 0.0002 | 1 | 0.0002 | 0.7029 |
|  | s(Altitude) | 0.0001 | 1 | 0.0006 | 0.3635 |
|  | s(PrimaryLandCover) | 5.4038 | 7 | 142611.1 | 0.0001 |
|  | s(Year__Fac) | 42.8692 | 46 | 51.7076 | < 0.0001 |

Table S2. The top ten most numerous moth species caught in each decade of the study, amounting to 19 species.

| **Binomial** | **Common name** |
| --- | --- |
| Agrotis exclamationis | Heart and Dart |
| Luperina testacea | Flounced Rustic |
| Orthosia gothica | Hebrew Character |
| Diarsia rubi | Small Square-Spot |
| Spilosoma lubricipeda | White Ermine |
| Epirrita dilutata | November Moth |
| Xanthorhoe montanata | Silver Ground Carpet |
| Eilema lurideola | Common Footman |
| Agrochola lychnidis | Beaded Chestnut |
| Xanthorhoe ferrugata | Dark-Barred Twin-Spot Carpet |
| Hydriomena furcata | July Highflyer |
| Idaea biselata | Small Fan-Footed Wave |
| Orthosia cruda | Small Quaker |
| Idaea aversata | Riband Wave |
| Xestia xanthographa | Square-Spot Rustic |
| Rivula sericealis | Straw Dot |
| Omphaloscelis lunosa | Lunar Underwing |
| Eilema griseola | Dingy Footman |
| Hypena proboscidalis | Snout |
